# Effect of EIT-guided PEEP titration on prognosis of patients with moderate to severe ARDS: study protocol for a multicenter randomized controlled trial

**DOI:** 10.1186/s13063-023-07280-6

**Published:** 2023-04-11

**Authors:** Xueyan Yuan, Rui Zhang, Yuxuan Wang, Dongyu Chen, Yali Chao, Jingyuan Xu, Lanqi Guo, Airan Liu, Jianfeng Xie, Chun Pan, Yi Yang, Haibo Qiu, Ling Liu

**Affiliations:** 1 Jiangsu Provincial Key Laboratory of Critical Care Medicine, Department of Critical Care Medicine, School of Medicine, Zhongda Hospital, Southeast University, Nanjing, 210009 Jiangsu China; 2 Department of Critical Care Medicine, The First People’s Hospital of Yancheng, Yancheng, 224000 Jiangsu China; 3 Department of Critical Care Medicine, The Affiliated Hospital of Xuzhou Medical University, Xuzhou, 320300 Jiangsu China

**Keywords:** Acute respiratory syndrome distress, Positive end-expiratory pressure, Electrical impedance tomography, Mechanical ventilation, Clinical trial

## Abstract

**Background:**

Acute respiratory syndrome distress (ARDS) is a clinical common syndrome with high mortality. Electrical impedance tomography (EIT)-guided positive end-expiratory pressure (PEEP) titration can achieve the compromise between lung overdistension and collapse which may minimize ventilator-induced lung injury in these patients. However, the effect of EIT-guided PEEP titration on the clinical outcomes remains unknown. The objective of this trial is to investigate the effects of EIT-guided PEEP titration on the clinical outcomes for moderate or severe ARDS, compared to the low fraction of inspired oxygen (FiO_2_)-PEEP table.

**Methods:**

This is a prospective, multicenter, single-blind, parallel-group, adaptive designed, randomized controlled trial (RCT) with intention-to-treat analysis. Adult patients with moderate to severe ARDS less than 72 h after diagnosis will be included in this study. Participants in the intervention group will receive PEEP titrated by EIT with a stepwise decrease PEEP trial, whereas participants in the control group will select PEEP based on the low FiO_2_-PEEP table. Other ventilator parameters will be set according to the ARDSNet strategy. Participants will be followed up until 28 days after enrollment. Three hundred seventy-six participants will be recruited based on a 15% decrease of 28-day mortality in the intervention group, with an interim analysis for sample size re-estimation and futility assessment being undertaken once 188 participants have been recruited. The primary outcome is 28-day mortality. The secondary outcomes include ventilator-free days and shock-free days at day 28, length of ICU and hospital stay, the rate of successful weaning, proportion requiring rescue therapies, compilations, respiratory variables, and Sequential Organ Failure Assessment (SOFA).

**Discussion:**

As a heterogeneous syndrome, ARDS has different responses to treatment and further results in different clinical outcomes. PEEP selection will depend on the properties of patients and can be individually achieved by EIT. This study will be the largest randomized trial to investigate thoroughly the effect of individual PEEP titrated by EIT in moderate to severe ARDS patients to date.

**Trial registration:**

ClinicalTrial.gov NCT05207202. First published on January 26, 2022.

## Administrative information

Note: the numbers in curly brackets in this protocol refer to SPIRIT checklist item numbers. The order of the items has been modified to group similar items together (see https://www.equator-network.org/reporting-guidelines/spirit-2013-statement-defining-standard-protocol-items-for-clinical-trials/).

**Table:** 

Title {1}	Effect of EIT-guided PEEP titration on prognosis of patients with moderate to severe ARDS: study protocol for a multicenter randomized controlled trial
Trial registration {2a and 2b}	ClinicalTrial.gov Identifier: NCT05207202. First published on January 26, 2022.
Protocol version {3}	Protocol Version 4, October 2022.
Funding {4}	This research is supported by National Key R&D Program of China (No. 2022YFC2504405), the Clinical Science and Technology Specific Projects of Jiangsu Province (BE2020786), the National Natural Science Foundation of China (81870066, 82270083), and the Second Level Talents of the “333 High Level Talents Training Project” in the sixth phase in Jiangsu (LGY2022025), Jiangsu Provincial Medical Key Laboratory (ZDXYS202205), and Draeger.
Author details {5a}	Xueyan Yuan^1^^†^, Rui Zhang^1^^†^, Yuxuan Wang^1^, Dongyu Chen^1, 2^, Yali Chao^1, 3^, Jingyuan Xu^1^, Lanqi Guo^1^, Airan Liu^1^, Jianfeng Xie^1^, Chun Pan^1^, Yi Yang^1^, Haibo Qiu^1^*, Ling Liu. ^1^Jiangsu Provincial Key Laboratory of Critical Care Medicine, Department of Critical Care Medicine, Zhongda Hospital, School of Medicine, Southeast University, Nanjing, 210009, Jiangsu, China. ^2^Department of Critical Care Medicine, The First people’s Hospital of Yancheng, Yancheng, 224000, Jiangsu, China. ^3^Department of Critical Care Medicine, The Affiliated Hospital of Xuzhou Medical University
Name and contact information for the trial sponsor {5b}	Jiangsu Provincial Key Laboratory of Critical Care Medicine, Department of Critical Care Medicine, Zhongda Hospital, School of Medicine, Southeast University. liulingdoctor@126.com.
Role of the sponsor {5c}	The sponsor had no influence on the research reported in this paper.

## Introduction

### Background and rationale {6a}

Acute respiratory distress syndrome (ARDS) is characterized by an acute inflammatory lung injury, associated with alveolar-capillary permeability, increased lung weight, and loss of aerated lung tissue [1]. This life-threatening condition clinically manifests as a rapid onset of severe hypoxemia secondary to many pulmonary or non-pulmonary insults and bilateral pulmonary infiltrates on chest imaging, which is associated with non-hydrostatic pulmonary edema [2, 3]. Despite considerable advances in clinical recognition and management of ARDS, it remains a leading cause of death in critically ill patients with a high mortality of approximately 40% [4]. Although mechanical ventilation (MV) is the cornerstone of the management of ARDS, it may aggravate ventilator-induced lung injury (VILI) [5].

ARDS, known as “baby lung,” presents a high degree of inhomogeneity and not inflated regions, mainly in the gravitationally dependent lung regions. Aerated, poorly aerated, and consolidated/collapsed regions indeed coexist in the ARDS lung parenchyma and the proportion of the recruitable regions is always less than 50% [6, 7]. The application of positive end-expiratory pressure (PEEP) to reduce the collapsed regions and further improve oxygenation has been largely accepted in clinical practice [8, 9]. Worth noting, the potential adverse effects of PEEP in mechanically ventilatory patients are not neglected, including circulatory depression and VILI [10]. The occurrence of VILI conceptually is associated with high local lung stress and parenchymal shear injury which may be caused by repetitive opening and closing of alveoli and distal small airways (“atelectrauma”) at low volume [5]. Experimental studies showed that atelectrauma is prominent in mechanically ventilatory patients with ARDS [11, 12]. Therefore, “open the lung and keep it open” was proposed based on the application of higher PEEP to prevent the intra-tidal collapse and decollapse [10, 13].

Although the selection of PEEP to minimize VILI has been investigated by numerous studies, substantial controversies still exist over the best strategy to set optimal PEEP for ARDS patients. Many different methods have been conducted in clinical trials to identify their effects on clinical outcomes. Setting PEEP based on the change in oxygenation is the most common approach and has been taken for the current standard of care in combination with low tidal volume (Vt) and plateau pressure (Pplat) ≤ 30 cm H_2_O. Brower et al. compared the effect of higher and lower PEEP in ARDS patients [14]. The setting of PEEP is based on the high or low FiO_2_-PEEP table in the study. The result showed that higher PEEP did not improve clinical outcomes. They supposed that it was possible that the beneficial effects of higher PEEP on reducing VILI from ventilation with nonaerated lung regions were offset by its adverse effects. In theory, atelectrauma may be mitigated by recruitment maneuvers (RM) to open collapsed lung tissue and high-level PEEP to prevent further collapse. The “lung open ventilation” (LOV) strategy was conducted to identify these effects [15]. In this study, the PEEP level was adjusted in terms of the oxygenation target after RM in the LOV strategy group, and followed by the ARDS network protocol in the control group. The trial demonstrated that there was no difference on mortality between the two groups. It is widely accepted that “optimal” PEEP may be the PEEP that best compromises between the overdistension of the aerated regions and the collapse of the recruited regions. “ExPress” study raised a ventilatory strategy using higher PEEP to increase recruitment while avoiding overdistension, and the results suggested that this method using lung mechanics was not effective in improving mortality [16]. “ART” study further demonstrated RM combined with PEEP titrated by the best respiratory-system compliance even increased 28-day mortality [17]. Due to the differences of thoracic compliance and intra-abdominal pressure among individuals, a given PEEP may contribute to different degrees of lung recruitment and distension in different patients. EPVent-2 trial compared esophageal pressure (P_ES_)-guided PEEP titration and high ARDSNet FiO_2_-PEEP table, and the results showed no significant difference in death and ventilator-free days (VFDs) was found [18].

Speculation on failing to show the benefits of higher PEEP is that none of these trials has been effective in assessing lung recruitability [19]. According to the morphological characteristics of lung lesions, ARDS can be classified into focal and non-focal as assessed by chest CT [20, 21]. Patients with focal ARDS have a lower amount of potentially recruitable lung and higher lung compliance. The application of higher PEEP may be more harmful than beneficial because it increases the inflation of aerated lung regions which in turn increases the stress and strain on these regions in these patients. Although CT-derived PEEP is physiologically considerable, the proof that is useful in guiding the management is lacking. “LIVE” study identified the effects of personalized MV tailored to lung morphology which was assessed by CT or chest x-ray, and the results demonstrated personalization of MV did not reduce mortality, a ventilator strategy misaligned with lung morphology, however, significantly increased the mortality in ARDS patients [20]. As its limitation, CT cannot be used at the bedside in clinical practice. So, it is not feasible for routine treatment in ARDS patients.

Electrical impedance tomography (EIT) is an imaging tool that allows individual, non-radiation, non-invasive, and real-time monitoring the regional ventilation distribution at the bedside [22]. EIT can identify the personalized PEEP by titrating PEEP to balance alveolar overdistension and recruitment [22, 23]. He et al. recently compared PEEP guided by EIT with low FiO_2_-PEEP table in ARDS patients [24]. They found that PEEP titrated by EIT had a better but insignificant survival. Potential explanation for this result may include the inclusion of mild and rapidly improving ARDS, short period of intervention, and effect of prone positioning. Considering these implications, further larger, multi-center randomized controlled trials are needed to identify the effects of PEEP titrated by EIT on the clinical outcomes.

### Objectives {7}

The primary objective of this trial is to determine whether EIT-guided PEEP titration can reduce 28-day mortality in moderate-to-severe ARDS patients ventilated with lung protective ventilation strategy compared to PEEP setting by low FiO_2_-PEEP table. Secondarily, we will further examine the effect of EIT-guided PEEP titration on the duration of MV, length of ICU and hospital stay, successful weaning, duration of shock-free day, compilations, and safety.

### Trial design {8}

This is a prospective, multicenter, single-blind, parallel-group, adaptive randomized controlled trial (RCT) with intention-to-treat analysis. The adaptive trial design with the interim analysis allows sample size re-estimation to assess the EIT-guided PEEP titration’s ability to improve clinical outcome when approximately 50% of the prespecified sample are enrolled. Only if the interim analyses were positive would the study continue until attaining the target size to evaluate 28-day mortality. The study will be conducted within a superiority framework. Patients with moderate or severe ARDS will be randomly assigned to ventilated in the lung protective ventilation strategy with PEEP titrated by EIT (EIT-PEEP strategy) or conventional approach (ARDSNet strategy) in 1:1 allocation ratio.

## Methods: participants, interventions, and outcomes

### Study setting {9}

The trial will be conducted in patients admitted to intensive care medicine over a period of approximately 3 years from 6 academic hospitals (Zhongda Hospital, School of Medicine, Southeast University; Zhongshan Hospital of Fudan University; The First Affiliated Hospital of Guangzhou Medical University; Beijing Tiantan Hospital, Capital Medical University; Renji Hospital, School of Medicine, Shanghai Jiao Tong University; Sichuan Provincial People’s Hospital, University of Electronic Science and Technology of China) in China. Participants will be identified by the study team.

### Eligibility criteria {10}

Patients who received invasive MV will be screened for eligibility. All eligible patients must fulfill the following inclusion criteria during screening and prior to enrolment into the study.

#### Inclusion criteria

1. Age ≥ 18 years
2. Moderate-to-severe ARDS, defined by the ARDS Definition Task Force in the Berlin definition (partial pressure of arterial oxygen [PaO_2_]:FiO_2_ ratio ≤ 200 mmHg with a PEEP ≥ 5 cmH_2_O) [25]
3. Diagnosis of ARDS less than 72 h

#### Exclusion criteria

All patients who meet any of the following criteria will be excluded at enrollment and randomization.

1. Expected to be mechanically ventilated for less than 48 h
2. Severe chronic respiratory diseases requiring long-term home oxygen therapy or noninvasive MV
3. Undrained pneumothorax or subcutaneous emphysema
4. Undergoing extracorporeal membrane oxygenation (ECMO) before enrollment
5. Contraindication to the use of EIT (pacemaker, automatic implantable cardioverter defibrillator, and implantable pumps)
6. Severe neuromuscular disease
7. Hemodynamic instability (> 30% increase in vasopressors within 6 h or norepinephrine > 0.5 µg/kg/min) [26]
8. Contraindications to hypercapnia, such as intracranial hypertension or acute coronary syndrome
9. Severe other organs dysfunction with a low expected survival (7 days) or palliative care
10. Solid organ or hematologic tumors with the expected survival time of less than 30 days
11. Participating in other clinical trials within 30 days
12. Pregnancy
13. Refusal to sign the informed consent

### Who will take informed consent? {26a}

A member of the study team will take consent prior to the start of the study activity. For patients who are unable to sign or initial the consent form, the consent form will be allowed to be signed and dated by his/her trustee or guardian.

### Additional consent provisions for collection and use of participant data and biological specimens {26b}

As a part of the consent form process, participants will be required to provide authorization for the extraction and use of their data. Participants will also be asked for permission for gathering de-identified information which may be used for ancillary studies. Biological samples obtained for further ancillary studies are not applicable in this study.

### Interventions

#### Explanation for the choice of comparators {6b}

**Fig. 1:**
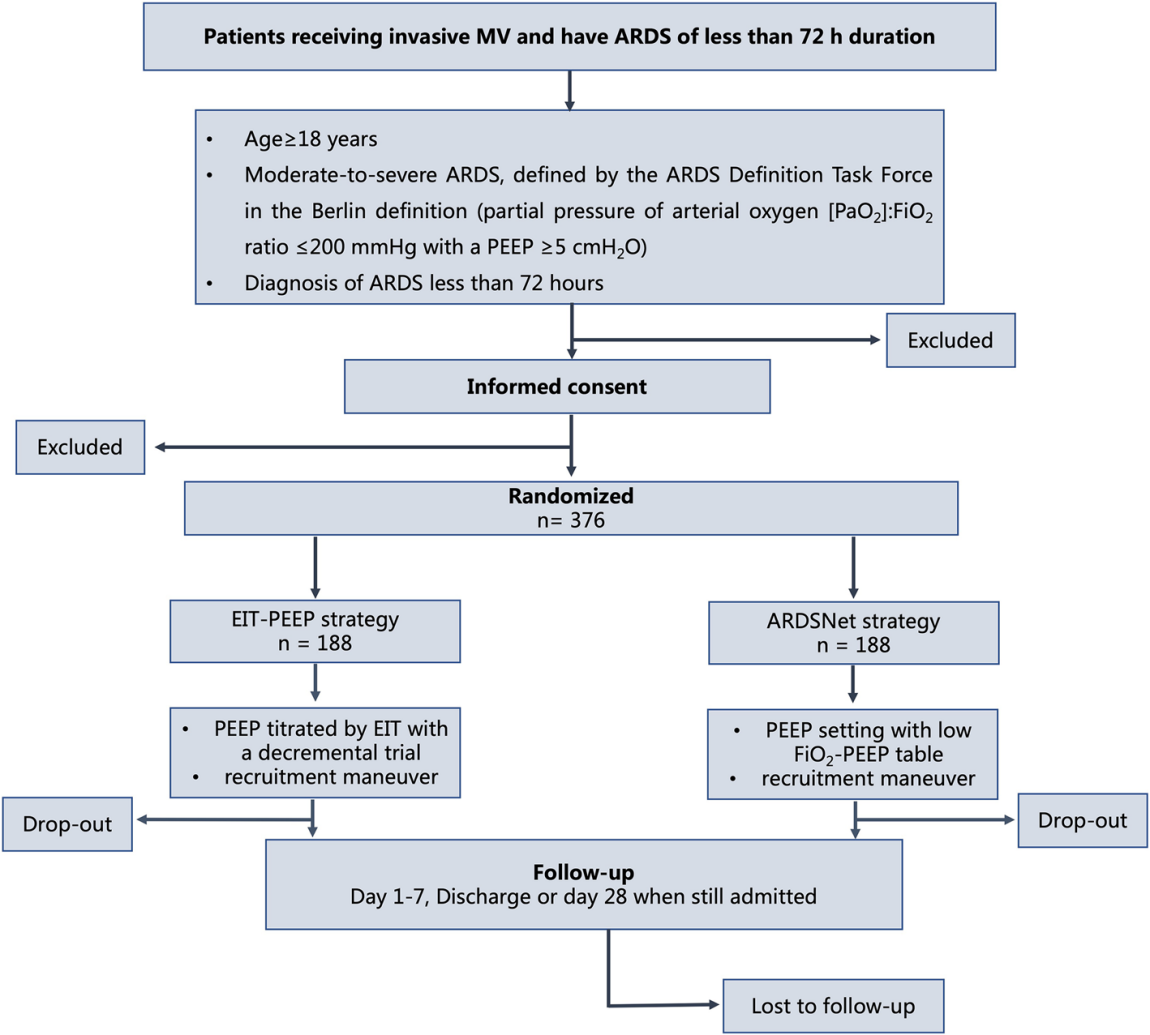
Study flow diagram. MV, mechanical ventilation; ARDS, acute respiratory distress syndrome; PEEP, positive end-expiratory pressure; EIT, electrical impedance tomography

EIT is increasingly used as a non-invasive image to assess lung ventilation and perfusion, which has been identified as a tool for PEEP setting in patients with ARDS. From the physiological standpoint, PEEP-guided EIT titration can achieve the compromission of alveolar overdistention and collapse. This may reduce VILI in these patients and further improve clinical outcomes. PEEP setting by EIT will be compared with the low FiO_2_-PEEP table, which is currently standard clinical practice. The study diagram is presented in Fig. 1.

#### Intervention description {11a}

The procedures used for the EIT-PEEP strategy and ARDSNet strategy groups are summarized in Table 1.

**Table 1:** Summary of mechanical ventilation characteristics in the EIT-PEEP and ARDSNet strategy

Procedure	EIT-PEEP strategy	ARDSNet strategy
Recruitment maneuver	Yes	Yes
Ventilation mode	Volume-control	Volume-control
Vt target	6–8 ml/kg of predicted body weight	6–8 ml/kg of predicted body weight
Plateau pressure	< 30 cm H2O	< 30 cm H_2_O
Respiratory rate	Set to attain target PaCO_2_ of 35–50 mmHg	Set to attain target PaCO_2_ of 35–50 mmHg
Oxygenation goal		
PaO_2_	55–80 mmHg	55–80 mmHg
SpO_2_	88–95%	88–95%
PEEP and FiO_2_ adjustment	Set guided by EIT	Set using low FiO_2−_ PEEP table
Criteria to initiate ventilator Weaning on pressure support mode	(1) The underlying condition that required MV improvement; (2) PaO_2_/FiO_2_ ≥ 200 mm Hg, PEEP ≤ 5 cm H_2_O, FiO_2_ ≤ 50%, RR < 35 breath/min; (3) hemodynamic stability; (4) no or low sedation	(1) The underlying condition that required MV improvement; (2) PaO_2_/FiO_2_ ≥ 200 mm Hg, PEEP ≤ 5 cm H_2_O, FiO_2_ ≤ 50%, RR < 35 breath/min; (3) hemodynamic stability; (4) no or low sedation

##### Ventilation settings

During this period, all patients will be fully sedated (with propofol, midazolam, and/or remifentanil), and even paralyzed (with a neuromuscular blocker) to prevent any spontaneous breathing. In addition, invasive arterial blood pressure is needed. Participants in both arms will be ventilated with volume-controlled mode. The initial Vt will be set to 6 ml/kg predicted body weight (PBW), and then adjusted to maintain Pplat less than 30 cmH_2_O according to ARDSNet protocol. The PBW is measured according to the formula:

$$\mathrm{Men}:\mathrm{ PBW}=50+0.91*\left(\mathrm{height }\left[\mathrm{cm}\right]-152.4\right)$$

$$\mathrm{Women}:\mathrm{ PBW}=45.5+0.91*\left(\mathrm{height }\left[\mathrm{cm}\right]-152.4\right)$$

The fraction of inspired oxygen (FiO_2_) will be adjusted for oxygen saturation (SpO_2_) of 88% to 95%, and PaO_2_ of 55 to 80 mmHg. The respiratory rate (RR) will be adjusted to maintain a partial pressure of carbon dioxide (PaCO_2_) between 35 and 50 mmHg based on arterial blood gases. The procedures that will be used for the EIT-PEEP group and ARDSNet group is summarized in Table 1.

##### PEEP setting in the EIT-PEEP group

A recruitment maneuver (RM) followed by the sustained inflation (SI) method with an airway pressure of 35–40 cmH_2_O for 30 s will be used to perform before the PEEP titration once or twice. Recruitment will be terminated if any of the following signs are observed: heart rate > 150 or < 60 breaths per minute (bpm); decrease of mean arterial blood pressure < 65 mmHg or decrease of systolic blood pressure < 90 mmHg; decrease of SpO_2_ < 88% for > 30 s; acute atrial fibrillation, atrial flutter, or ventricular tachycardia.

**Fig. 2:**
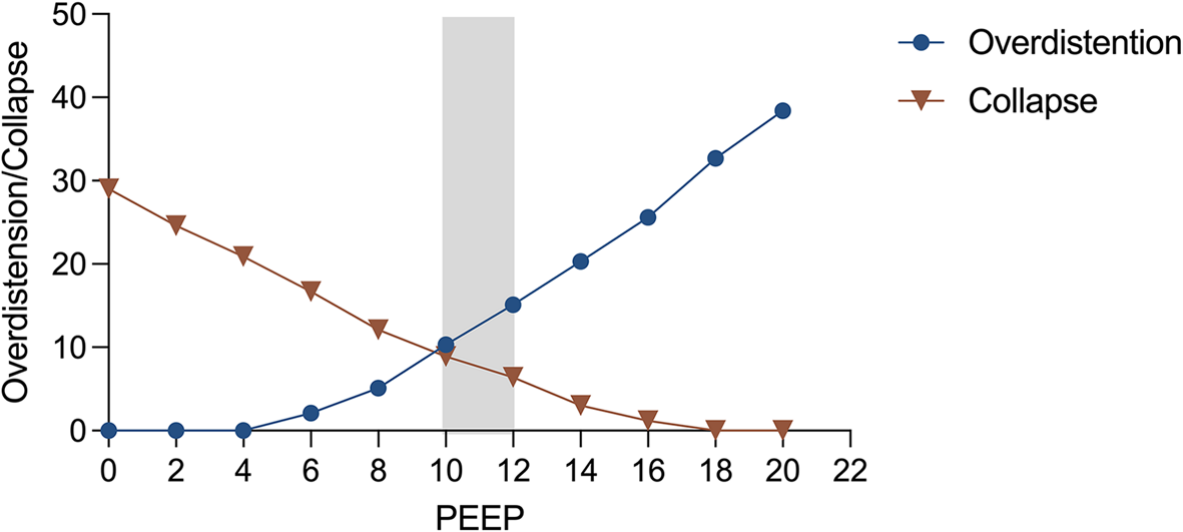
Setting positive end-expiratory pressure (PEEP) based on electrical impedance tomography. PEEP, positive end-expiratory pressure

EIT-guided PEEP titration will be performed with a decremental trial at the enrollment. Right after completing RM, PEEP will be set to 20 cmH_2_O and then reduced in steps of 2 cmH_2_O from 20 to zero every 2 min. The EIT device provides the percentages of alveolar overdistention and collapses at each PEEP level. The best compromised PEEP is defined as the PEEP level above the intersection of curves representing relative alveolar overdistention and collapse (Fig. 2). Once the best compromised PEEP is identified, the lung will be again recruited. PEEP will then be set to the best compromised PEEP and kept to the next PEEP titration. In addition, FiO_2_ will be reduced by 5% step if the PaO_2_ > 80 mmHg. PEEP will be titrated every 24 h between 8:00 and 10:00 AM. Moreover, PEEP titrated by EIT needs to be repeated within 2 h after prone positioning. The process will exist for 7 days unless the selected PEEP is less than 5 mmHg. Participants randomized to the EIT-PEEP group will be remained in the protocol for up to 7 days. Thereafter, they will receive MV according to the ARDSNet recommendation guidelines.

##### PEEP setting in ARDSNet strategy

RM followed by SI method with an airway pressure of 35–40 cmH_2_O for 30 s will be used to perform before the PEEP setting once or twice. PEEP will be subsequently set according to the low FiO_2_-PEEP table (Table 2) to keep the oxygenation goals: SpO_2_ between 88 and 95%, and PaO_2_ between 55 and 80 mmHg (Table 2). PEEP will be titrated once daily and maintained until the next PEEP titration (titrated every 24 h, 8:00–10:00 AM) for 7 days. Patients randomized to the ARDSNet group will be remained in the protocol for up to 7 days. Thereafter, they will receive MV according to the ARDSNet recommendation guidelines.

**Table 2:** Low FiO_2_-PEEP table

FiO_2_	30%	40%	40%	50%	50%	60%	70%	70%	70%	80%	90%	90%	90%	100%
PEEP	5	5	8	8	10	10	10	12	14	14	14	16	18	18–24

### Criteria for discontinuing or modifying allocated interventions {11b}

Participants have the right to discontinue the intervention voluntarily at any time and the reason is not required to provide. When a participant withdraws from the trial due to an adverse event or treatment failure, the study staff should take the necessary measures to minimize the adverse effects in accordance with the patient's condition. The follow-up will proceed normally, and relevant data should be properly preserved, not only for archiving, but also for statistics of full analysis set (FAS).

Participants will be withdrawn from the trial which is determined by the investigator when any of the following criteria is met: (1) combined with other diseases that affected the judgment of efficacy and safety during the trial; (2) seriously violated eligibility criteria or met the inclusion criteria but not treated after randomization.

When a participant withdraws from the trial, the study staff should take measures to complete the last test as far as possible for the analysis of the efficacy and safety. The necessary measures to minimize adverse effects will be applied when patients withdraw consent to the study. For all withdrawal cases, the study conclusion sheet and the reason and circumstances for the withdrawal should be filled in the research medical record.

### Strategies to improve adherence to interventions {11c}

All study staff will receive protocol and device training before they can obtain the delegation and training logs to ensure protocol adherence. Once the patient is enrolled, the study protocol will be laminated and pace by the study team at the bedside, and the registered nurse will be informed. Meanwhile, a customized card containing the study protocol will be placed at the bedside of the participant.

### Relevant concomitant care permitted or prohibited during the trial {11d}

Routine standard of care is allowed throughout the study, whereas the randomized intervention will be executed at least 16 h per day. All clinical practice will be consistent with the pragmatic study design. So, there are no restrictions on concomitant care.

### Provisions for post-trial care {30}

The participants will complete the study follow-ups to 28 days, with serious adverse events being recorded during this period. After participation in the trial, participants will continue the routine standard of care.

### Outcomes {12}

#### Primary outcome

- 28-day mortality (participant will be a follow-up to 28 days)

#### Secondary outcomes

- Mechanical ventilation-free from day 1 to 28 (VFDs, defined as the number of days between successful weaning from MV and day 28 after study enrollment. For patients ventilated for 28 days or more and for patients who die, the VFDs are 0.)
- Shock-free days (no vasopressor requirement) from day 1 to 28
- Length of ICU stay
- Length of hospital stay
- The rate of successful weaning (defined as the absence of the requirement for ventilatory support, without reintubation, a cardiac arrest event, or mortality within 48 h after extubating or withdrawal)
- Proportion requiring rescue therapies: (i) neuromuscular blocker, (ii) prone position, (iii) high-frequency oscillatory ventilation, and (iv) ECMO
- Compilations: pneumothorax, pneumomediastinum, hemodynamic adverse events, and time of tracheotomy (defined as days from baseline to tracheotomy)
- Respiratory variables: blood gas analysis, SpO_2_, and RR at enrollment (D0), 24 h after enrollment (D1), 48 h after enrollment (D2), 72 h after enrollment (D3), 7 days after enrollment (D7)/last day of invasive ventilatory support/ICU discharge.
- Hemodynamic variables: mean blood pressure, heart rate, and central venous pressure (CVP) at baseline, D1, D2, D3, and D7/ last day of invasive ventilatory support/ICU discharge
- Respiratory mechanics: ventilator mode, Ppeak, Pplat, flow, and respiratory compliance at baseline, D1, D2, D3, D4, D5, D6, and D7/ last day of invasive ventilatory support/ICU discharge
- Sequential Organ Failure Assessment (SOFA) at D0
- Adverse events

### Participant timeline {13}

The participant timeline is presented in Table 3.

**Table 3:**
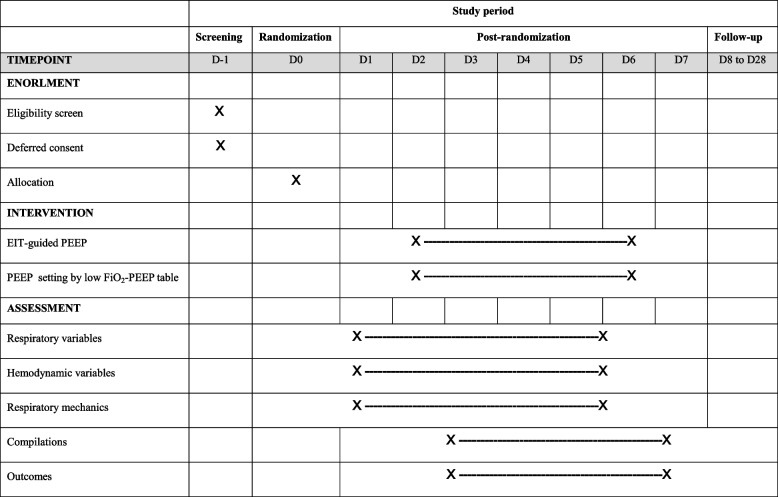
Participant timeline

### Sample size {14}

The primary outcome of this study is 28-day mortality with EIT-guided PEEP titration (EIT-PEEP group) as the intervention group and PEEP setting based on the low FiO_2_-PEEP table (ARDSNet group) as the control group. According to previous study, it is anticipated that the 28-day mortality of the FiO_2_-PEEP group is 39% [27], with a 15% decrease in the EIT-PEEP group [28]. We hypothesize that EIT-guided PEEP titration will improve 28-day mortality, with a probability of α = 0.025 (single-side) to allow for type I error, and power 0.85. A seqdesign procedure in SAS software is performed to calculate the sample size and determine that a sample size of 169 patients per arm is required. A final sample size of 188 per arm accounts for an additional 10% dropout rate in case of follow-up.

### Recruitment {15}

Patients’ recruitment is currently conducted in the six centers. The estimated recruitment in each center is as follows: Zhongda Hospital, School of Medicine, Southeast University (5–6 participants); Zhongshan Hospital of Fudan University (4–5 participants); The First Affiliated Hospital of Guangzhou Medical University (3–4 participants); Beijing Tiantan Hospital, Capital Medical University (3–4 participants); Renji Hospital, School of Medicine, Shanghai Jiao Tong University (1–2 participants); Sichuan Provincial People’s Hospital, University of Electronic Science and Technology of China (3–4 participants). Recruitment started in February 2022 in one center. As of February 21, 2023, we had already enrolled 139 patients. There is no specific strategy to promote the rate of patients’ recruitment.

### Assignment of interventions: allocation

#### Sequence generation {16a}

Patients will be block-randomized in a 1:1 ratio to the EIT-PEEP strategy or ARDSNet strategy. The randomization list was generated by a statistician in the study team according to the computer-generated number and it was then sealed.

#### Concealment mechanism {16b}

An online computer-generated system will conceal the randomization sequence until planned initiation of the study intervention.

#### Implementation {16c}

The allocation sequence was computer generated by a sealed envelope and submitted to the clinical research unit for storage. The method of random number generation, process, group setting, and grouping results will be recorded and explained for checking when necessary. After taking informed consent, confirmation of eligibility, and completion of baseline assessments, the study site staff will randomize the patients using sealed envelopes.

### Assignment of interventions: blinding

#### Who will be blinded {17a}

Blinding of trial participants is not necessary since the intervention will be administered to critically ill patients on invasive MV who are mostly sedated. By the nature of the intervention, the treatment allocation assigned to each participant is not possible to blind to the study staff. Screening, enrollment, and random code allocation of patients are performed by the study staff. Also, the name and random number of the randomized patients were recorded and saved by the study staff.

#### Procedure for unblinding if needed {17b}

No procedure for unblinding will be necessary as neither the study staff nor the participant will be blinded to treatment allocation.

### Data collection and management

#### Plans for assessment and collection of outcomes {18a}

The patients will be visited daily between days 1 and 7 after enrollment, on ICU discharge, and 28 days after enrollment. Study follow-up and patients’ data are recorded below.

##### Baseline

Baseline data will be collected once the study staff takes valid informed consent, including patients’ demographics (age, gender, height, weight); physical status at ICU admission (diagnosis, date of ICU admission, acute physiology, and chronic health evaluation II (APACHE II) score, SOFA, causes of ARDS, days of intubation prior randomization, timing of ARDS onset); past disease history; and lung recruitability (assessing by recruitment-to-inflation ratio).

##### At the time of randomization (D0)

The following data will be collected: respiratory variables (SpO_2_, ventilator mode, Ppeak (peak airway pressure), Pplat, flow, PEEP, total RR, FiO_2_, Arteria blood gas analysis (arteria pH, PaO_2_, PaCO_2_, PaO_2_/FiO_2_); hemodynamic variables (heart rate, mean arterial pressure, central venous pressure (CVP), use of vasopressors); blood chemistry.

##### Period of intervention (1-day follow-up to 7-day follow-up)

Data during the period of intervention will be collected, including respiratory variables (SpO_2_, ventilator mode, Ppeak, Pplat, flow, PEEP, total RR, FiO_2_, Arteria blood gas analysis (arteria pH, PaO_2_, PaCO_2_, PaO_2_/FiO_2_) (D1, D2, D3, D7)); hemodynamic variables (heart rate, mean arterial pressure, CVP, use of vasopressors (up to daily)); blood chemistry (up to daily); need for rescue therapies (up to daily); adverse events (up to daily).

##### Primary and secondary outcome data collection

Assessment of the primary outcome will be conducted at the 28 days of follow-up (study protocol initiated).

Data on the secondary outcome will be collected at discharge from ICU and hospital and 28 days after the initiation of the study protocol.

##### Plans to promote participant retention and complete follow-up {18b}

All participants will be informed of 28 days of follow-up and phone calls to ensure the completion of surveys at consent. Participants choosing to withdraw consent to the study will be encouraged to continue with the follow-up of the trial for the evaluation of efficacy and safety. The reasons and circumstances for discontinuing the study will be recorded.

##### Data management {19}

Data will be collected by paper case report form (CRF) and then stored as electronic data using the REDCap system with access limited to the study staff and supervisors. Range checks for data values are built into the system to ensure data quality. The data will be pseudonymized and all participants will be assigned a unique trial identification code. All study material will be kept in a special filing cabinet for information security and later traceability.

##### Confidentiality {27}

Participants’ data will be considered as strictly confidential. All study staff must follow the requirements of all data and privacy laws relevant to the jurisdiction with respect to the collection, storage, processing, and disclosure of personal information.

##### Plans for collection, laboratory evaluation, and storage of biological specimens for genetic or molecular analysis in this trial/future use {33}

This trial does not contain biological specimens for genetic or molecular analysis. All laboratory results collected from participants are obtained from clinical routine blood tests.

### Statistical methods

#### Statistical methods for primary and secondary outcomes {20a}

##### Statistical analysis

Data will be collected using a standardized CRF, and the statistical analysis will base on an intention-to-treat basis. SAS 9.4 statistical software will be used for analysis.

Continuous data will be reported as mean (standard deviation) or median (interquartile range) depending on the nature and distribution. For data normally distribution, the Student *t* test will be used for comparison. The Mann–Whitney *U* rank test will be used for skewed distributed data. Categorical data will be reported as counts and percentages. Group comparisons will be made using the chi-square test, Fisher’s exact test, or Wilcoxon’s signed-rank test for categorical variables, whenever appropriate.

The primary outcome (28-day mortality) will be assessed using Kaplan–Meier curves and Cox proportional hazard models, without adjustment for other covariates. Treatment effects on VFDs at day 28, shock-free days at day 28, length of ICU stay, length of hospital stay, SOFA, and respiratory variables will be analyzed using *t* test. The rate of successful weaning, proportion requiring rescue therapies, and compilations will be assessed using the chi-square test or Fisher’s exact test. All tests will be two-tailed, and *P* value < 0.05 will be considered significant.

##### Interim analysis {21b}

The interim analysis will be performed after the recruitment of approximately 50% of the sample to assess the effects on clinical outcomes. The conditional power (Cp) will be calculated when 50% of patients complete the follow-up. The sample size will increase if necessary and the re-estimation of the sample size will base on the Cp value. The interim analysis will not be performed again if the sample size has increased. The independent data monitoring committee (IDMC) will be established to operate and make decisions for the interim analysis.

##### Methods for additional analyses (e.g., subgroup analyses) {20b}

Treatment effects on 28-day mortality will be analyzed according to the following subgroups: (1) PaO_2_/FiO_2_; (2) SOFA; (3) respiratory compliance; (4) lung recruitability; (5) pulmonary ARDS *vs.* extrapulmonary ARDS; (6) vasopressors *vs.* non-vasopressors; (7) prone position *vs.* non-prone position; (8) ECMO *vs.* non-ECMO.

##### Methods in analysis to handle protocol non-adherence and any statistical methods to handle missing data {20c}

Both intention-to-treat and per-protocol analyses will be reported to assess the robustness of the results at the final analysis. In case of missing data, the reason and mechanism for missing data will be explored. For missing data greater than 20%, multiple imputation may be considered as a sensitivity analysis to evaluate the treatment effect and associated standard error as appropriate.

##### Plans to give access to the full protocol, participant-level data, and statistical code {31c}

All of the full protocol, participant-level data, and de-identifiable data will be made available from the corresponding author if requested by the members of the trial with reasonable cause.

### Oversight and monitoring

#### Composition of the coordinating center and trial steering committee {5d}

The coordinating center is Zhongda Hospital, School of Medicine, Southeast University. The trial steering committee (TSC) is responsible for the overall study supervision and assisting in reviewing and approving any modifications to the study protocol. The TSC is composed of investigators trained in designing and conducting.randomized clinical trials, clinicians, and statisticians.

#### Composition of the data monitoring committee, its role, and reporting structure {21a}

IDMC is set up to provide recommendations for the TSC of continuing the study as planned or discontinuing the recruitment based on interim analyses. The IDMC is composed of independent expert statisticians and clinicians, so as to ensure the blinding of the trial and the objectivity of decision-making. The statistician will provide the interim reports for the DMEC. The confidentiality of trial results is ensured throughout the process. Only the IDMC has assessed to unblind the results before the end of the trial.

#### Adverse event reporting and harms {22}

Any unexpected medical events occurring in the participant during the intervention period will be considered as an adverse event (AE). Serious adverse events (SAE) are defined as adverse events (including death, life-threatening, permanent or severe disability or loss of function, and need for or prolonged hospitalization) that occur following the trial treatment. All AE will be recorded in CRF, and SAE will be reported to the principal investigators as soon as possible. Once AE is found, the investigators will assess the association between intervention and AE and decide whether to stop the intervention. The patients who discontinued the intervention because of AE will be followed up.

#### Frequency and plans for auditing trial conduct {23}

The sponsor will perform monitoring for each site to ensure the study quality, including the accuracy of data entered in REDCap, as well as to assess the occurrence of risks and benefits and detect evidence of achieving the primary objective of the study.

#### Plans for communication of important protocol amendments to relevant parties (e.g., trial participants, ethical committees) {25}

Any change in the study will generate synchronous protocol amendments, which will be submitted for approval to the Ethics Committee/institutional review board for filing in a timely manner. The changes will only be implemented after approval by the ethical committee. Once approved, the amendments will be circulated to the other study site and ClinicalTrials.gov will be synchronously updated about any major changes; if necessary, protocol training will be provided for the amendments by the study team.

#### Dissemination plans {31a}

Once available, the results of this research will be presented at conferences, published in scientific journals, and shared with the practice. The primary outcome of the study will be published as the first article and additional results derived from the data could be published in separate articles. If the results are favorable to the intervention, we will share details of the intervention with other healthcare organizations.

## Discussion

In patients with moderate to severe ARDS, the application of PEEP is the most widely accepted approach to improve alveolar recruitment. However, the strategy of the PEEP setting is substantially controversial in these patients. Hence, evidence from well-designed and conducted trials is needed to resolve this problem. This trial is designed to evaluate the effects of an individualized PEEP setting guided by EIT on the clinical outcomes in patients with moderate or severe ARDS, compared with a PEEP setting with a low FiO_2_-PEEP table.

ARDS is a heterogeneous syndrome because of the different etiology, time of onset, activation of inflammation, respiratory mechanics, and lung recruitability [2, 3]. Different subphenotypes have different responses to the ventilatory treatment, which further result in distinct outcomes. Due to the substantial heterogeneity of ARDS, one fixed PEEP does not fit all these patients. The PEEP selection should be an individual process depending on the properties of the patient. Individualized PEEP, as measured with EIT at the lowest relative alveolar overdistention and collapse, may improve ventilation inhomogeneity. Zhao et al. confirmed that the individual PEEP setting using a standardized incremental PEEP trial with EIT could achieve the most homogeneous ventilation in the lung [29]. A recent study comparing the two different methods of PEEP selection showed that PEEP setting guided by EIT could facilitate more homogeneous distribution of ventilation and moderate dorsal hypoventilated units [30]. However, there is limited information on the effect of PEEP setting guided by EIT in the outcomes of ARDS patients.

This study was planned to be the largest randomized trial including moderate to severe ARDS patients to investigate thoroughly the effect of individual PEEP selection by EIT to date. It will provide more deep insight into the PEEP selection for clinicians in clinical practice.

## Trial status

The trial is currently ongoing in 6 sites (Zhongda Hospital, School of Medicine, Southeast University; Zhongshan Hospital of Fudan University; The First Affiliated Hospital of Guangzhou Medical University; Beijing Tiantan Hospital, Capital Medical University; Renji Hospital, School of Medicine, Shanghai Jiao Tong University; Sichuan Provincial People's Hospital, University of Electronic Science and Technology of China) in China. Enrollment started in February 2022 in one site. Now, all 6 sites are actively screening for patients, and the remaining are undergoing REB evaluation. As of February 21, 2023, we had already enrolled 139 patients. The protocol version is 4.0 (8th October 2022).

## Data Availability

Any data collected in this study can be obtained from the corresponding author with a reasonable request.

## Abbreviations

ARDS: Acute respiratory distress syndrome
EIT: Electrical impedance tomography
PEEP: Positive end-expiratory pressure
FiO_2_: Fraction of inspired oxygen
RCT: Randomized controlled trial
SOFA: Sequential Organ Failure Assessment
MV: Mechanical ventilation
VILI: Ventilator-induced lung injury
Vt: Tidal volume
Pplat: Plateau pressure
RM: Recruitment maneuvers
LOV: Lung open ventilation
P_ES_: Esophageal pressure
VFDs: Ventilator-free days
PaO_2_: Partial pressure of arterial oxygen
ECMO: Extracorporeal membrane oxygenation
PBW: Predicted body weight
SpO_2_: Oxygen saturation
RR: Respiratory rate
PaCO_2_: Partial pressure of carbon dioxide
SI: Sustained inflation
FAS: Full analysis set
CVP: Central venous pressure
APACHE II: Acute physiology and chronic health evaluation II
CRF: Case report form
IDMC: Independent data monitoring committee
TSC: Trial steering committee
AE: Adverse event
SAE: Serious adverse events
